# QTL-seq for the identification of candidate genes for days to flowering and leaf shape in pigeonpea

**DOI:** 10.1038/s41437-021-00486-x

**Published:** 2022-01-12

**Authors:** Vikas Singh, Pallavi Sinha, Jimmy Obala, Aamir W. Khan, Annapurna Chitikineni, Rachit K. Saxena, Rajeev K. Varshney

**Affiliations:** 1 Center of Excellence in Genomics & Systems Biology, International Crops Research Institute for the Semi-Arid Tropics (ICRISAT), Hyderabad, 502 324 India; 2 International Rice Research Institute (IRRI), South-Asia Hub, ICRISAT, Hyderabad, India; 3 Department of Science, Lira University, Lira, Uganda; 4 Gujarat Biotechnology University (GBU), Gandhinagar, 382011 Gujarat India; 5 State Agricultural Biotechnology Centre, Centre for Crop and Food Innovation, Food Futures Institute, Murdoch University, Murdoch, WA WA6150 Australia

**Keywords:** Genetic association study, Plant hybridization

## Abstract

To identify genomic segments associated with days to flowering (DF) and leaf shape in pigeonpea, QTL-seq approach has been used in the present study. Genome-wide SNP profiling of extreme phenotypic bulks was conducted for both the traits from the segregating population (F_2_) derived from the cross combination- ICP 5529 × ICP 11605. A total of 126.63 million paired-end (PE) whole-genome resequencing data were generated for five samples, including one parent ICP 5529 (obcordate leaf and late-flowering plant), early and late flowering pools (EF and LF) and obcordate and lanceolate leaf shape pools (OLF and LLS). The QTL-seq identified two significant genomic regions, one on CcLG03 (1.58 Mb region spanned from 19.22 to 20.80 Mb interval) for days to flowering (LF and EF pools) and another on CcLG08 (2.19 Mb region spanned from 6.69 to 8.88 Mb interval) for OLF and LLF pools, respectively. Analysis of genomic regions associated SNPs with days to flowering and leaf shape revealed 5 genic SNPs present in the unique regions. The identified genomic regions for days to flowering were also validated with the genotyping-by-sequencing based classical QTL mapping method. A comparative analysis of the identified seven genes associated with days to flowering on 12 *Fabaceae* genomes, showed synteny with 9 genomes. A total of 153 genes were identified through the synteny analysis ranging from 13 to 36. This study demonstrates the usefulness of QTL-seq approach in precise identification of candidate gene(s) for days to flowering and leaf shape which can be deployed for pigeonpea improvement.

## Abstract (web summary)

QTL-seq approach was utilized for mapping of genomic regions/genes associated with days to flowering and leaf shape in pigeonpea. Analysis of genomic regions and associated SNPs with days to flowering and leaf shape revealed 1 and 4 non-synonymous SNPs, respectively. The study demonstrated sequencing-based trait mapping approach can accelerate trait mapping of the targeted traits.

## Introduction

Pigeonpea [*Cajanus cajan* (L.)] is a protein-rich food legume that serves the dietary needs of more than a billion people in the developing world (Valenzuela 2011). Multiple uses of pigeonpea as food, livestock feed/fodder and domestic firewood make it a sustainable crop of small-holding farmers in the marginal and risk-prone rainfed conditions (Saxena 2008). Development and adoption of improved varieties with higher yield will enhance the availability of plant-based protein for per capita consumption, thereby reducing the number of malnourished people across the world, especially in developing countries.

In pigeonpea, genomics approaches are being deployed to identify genomic regions that confer resistance/tolerance for different stresses. Both biparental mapping and association mapping approaches have been utilized to dissect complex traits in pigeonpea (Bohra et al. 2020). With the advantage of Next Generation Sequencing (NGS) technologies and availability of the pigeonpea reference genome sequence (Varshney et al. 2012), trait mapping approaches have mainly focused on mapping biotic stresses like sterility mosaic disease (SMD) and *Fusarium* wilt (FW) (Singh et al. 2016a, 2016b; Singh et al. 2017b; Saxena et al. 2017a; 2017b), abiotic stress like drought (Sinha et al. 2015), a marker for A4-derived CMS (Sinha et al. 2016), growth habit (Saxena et al. 2017c), A4-CMS restoration (Saxena et al. 2018), cleistogamous flower, shriveled seed and seed size (Yadav et al. 2019), seed protein content (Obala et al. 2019; Obala et al. 2020).

For developing new plant types that can suit various production niches, crop diversification with the development of photo-insensitive early maturing pigeonpea cultivars is a prerequisite (Saxena et al. 2019a). The first spontaneous mutant early maturing cultivar was detected in 1953 in a farmer’s field. This triggered breeding of early maturing varieties, and subsequently, pigeonpea cultivars varying in maturity periods were bred (Saxena et al. 2019b). Since then, several early maturing cultivars have been bred in different parts of the world. Similarly, leaf shape is another morphological marker (naked eye polymorphism) in cytoplasmic male sterile (CMS) and the corresponding maintainer lines to track purity of the inbred lines and corresponding hybrids for large scale commercial hybrid seed production (Saxena et al. 2011b). It was noted that the obcordate leaf morphological marker is present in accession ICP 5529, which can be easily assessed visually in about 6 weeks after sowing.

Availability of pigeonpea genome assembly along with advances in NGS provides an opportunity to develop genomics tools and technologies for the mapping of agronomically important traits such as days to first flowering and leaf shape in pigeonpea. QTL identification using whole-genome resequencing of two DNA bulks of progeny showing extreme phenotype (QTL-seq) is an emerging technology that enables locating and refining candidate genomic regions more efficiently compared to traditional QTL mapping approaches (Takagi et al. 2013). As the QTL-seq technique is independent of DNA marker development and genotyping the whole population, it is a time-saving and cost-effective procedure as compared to the conventional QTL analysis. The QTL-seq approach has a wide applicability in QTL identification in many agronomically important crops like rice (Takagi et al. 2013), chickpea (Das et al. 2015; Singh et al. 2016b), groundnut (Pandey et al. 2017; Kumar et al. 2020), pigeonpea (Singh et al. 2016a; Singh et al. 2017b), cucumber (Lu et al. 2014), and tomato (Illa**-**Berenguer et al. 2015).

With the objective of identifying candidate genomic regions responsible for days to flowering (DF) and leaf shape in pigeonpea, QTL-seq approach was adopted. We were able to precisely localize genomic regions for two target traits and identify nine genic SNPs in seven candidate genes for DF and 39 genic SNPs in 20 candidate genes for leaf shape through QTL-seq approach. The involvement of candidate genes was further validated through co-segregation analysis in the entire F_2_ population derived from ICP 5529 × ICP 11605 through the genotyping-by-sequencing (GBS) based approach.

## Materials and methods

### Plant materials

One F_2_ segregating population comprising 179 lines developed from a late duration (105 DF) and obcordate leaf shape, ICP 5529 and an early duration (67 DF) lanceolate leaf (normal) genotype, ICP 11605 was used in the present study (Obala et al. 2019). For trait evaluation, the parents and seeds of the mapping population were sown under field conditions. Sowing was done in 4 m long rows spaced 75 cm apart and 30 cm plant to plant distance within a row. Plot sizes were two rows for each of the two parents and 25–28 rows in the F_2_s. All cultural practices were carried out. Days to flowering (DF) were recorded for individual plants as number of days to first flowering after sowing, whereas leaf shape data was recorded 6 weeks after sowing.

### Construction of pools

Extreme bulks were prepared for days to first flowering and leaf shape traits based on precise phenotyping data obtained for F_2_ population. For developing the extreme bulks for each trait, 15 F_2_s with high mean phenotypic values and 15 F_2_s with low mean phenotypic values were selected in the case of days to first flowering. For leaf shape, 15 F_2_s of lanceolate leaf shape (LLS) (normal leaf) and 15 F_2_s with obcordate leaf shape were selected for the preparation of the bulks. The equimolar concentration of DNA from 15 F_2_s with high mean phenotypic values were pooled together as one bulk, and similarly, DNA from low mean phenotypic values were pooled together as another bulk. Thus, two bulks of pooled DNA each for both traits (DF and leaf shape) were used for library preparation and sequencing.

### Construction of libraries and Illumina sequencing

A total of five genomic libraries (four from extreme bulks mentioned above and one from ICP 5529 parent) were prepared using TruSeq DNA Sample Prep kit LT, (set A) FC-121-2001. Two microgram of DNA from each sample was sheared using diagenode Bioruptor^®^ NGS, end-repaired and adapter-ligated. Size selection of libraries was performed using 2% agarose gel to get a target insert size of 500–600 bp and purified for further analysis. Further, the libraries were enriched using adaptor compatible PCR primers. The size distribution of amplified DNA libraries was checked on an Agilent Technologies 2100 Bioanalyzer using a High Sensitivity chip. The DNA libraries were sequenced on Illumina HiSeq platform with HiSeq Reagent Kit v2 (500-cycles) to generate 250 base paired-end (PE) reads.

### Construction of reference-guided assembly

The statistics of generated sequencing reads were estimated using the raspberry tool of NGS-QCbox (Katta et al. 2015). Furthermore, QTL-seq pipeline (http://genome-e.ibrc.or.jp/home/bioinformatics-team/mutmap, developed by Iwate Biotechnology Research Center, Japan) was used for calculating SNP-index. Briefly, the cleaned reads of ICP 5529 were first aligned to the reference genome (Varshney et al. 2012) using inbuilt BWA aligner (Li and Durbin 2009). Coval was used for post-processing and filtering of the alignment files (Kosugi et al. 2013). The variants called for ICP 5529 were then used to develop a reference-guided assembly of ICP 5529 by substituting the bases with confidence variants called in the genome. The reads from extreme bulks for both the traits were then aligned, and variants were called for both the bulks against the developed assembly.

### Calculation of SNP-index

SNP-index for each SNP position was calculated for both the bulks as per Abe et al. (2012) using the formula: SNP-index (at a position) = Count of alternate base/ Count of reads aligned. The positions with read depth < 7 in both the bulks and SNP-index < 0.3 in either of the bulks were filtered out, and SNPs with homozygous alleles in both the bulks were used for ∆SNP-index calculation. ∆SNP-index can be calculated by subtracting the SNP-index of low bulk from SNP-index of high bulk. Only SNP positions with ∆SNP-index = −1 (i.e., the allele called in high trait value-bulk was the same as that of the resistant parent while contrastingly different in low trait value-bulk) were considered as the causal SNPs responsible for the trait of interest. The possible effects of the identified SNPs were inferred using SnpEff v3.0 open-source program (Cingolani et al. 2012).

## Results

### Construction of extreme bulks for days to flowering and leaf shape

Based on phenotyping data generated on F_2_s derived from a crossing combination ICP 5529 × ICP 11605, two extreme bulks each for DF (early and late) and leaf shape (normal lanceolate and obcordate types) were prepared and subjected to the QTL-seq pipeline as shown in Figure S1 and Table S1. Phenotyping data on DF in F_2_s showed a variation from 65 to 102 days in comparison to parental lines (105 days of ICP 5529 and 67 days of ICP 11605). The absolute difference between the parental lines was 38 days. Shapiro–Wilk test showed that distribution for DF was significantly different from a Gaussian distribution (*P* ≤ 0.05) (Table S2).

In the case of leaf shape trait, all the F_1_s from the cross had normal lanceolate leaves suggesting recessive nature of the obcordate leaf shape, while in the F_2_ population, segregation for the same trait fitted well to the expected ratio of 3:1 (3 lanceolate: 1 obcordate leaf) (Table S3). Based on the phenotyping data, 15 F_2_s with early DF (65–66 days, early flowering pool, EF) and 15 F_2_s with late DF (92–102 days, late-flowering pool, LF) were selected to prepare two extreme bulks (Table S4). For leaf shape 15 F_2_s with LLS (lanceolate leaf pool, LLS pool) and 15 F_2_s with obcordate leaf shape (obcordate leaf shape pool, OLS pool) were selected to prepare extreme bulks (Table S5).

### Whole genome sequencing and mapping of reads

Five genomic libraries (two for DF bulks, two for leaf shape bulks, and one for ICP 5529, the obcordate leaf shape parent) were constructed and subjected to whole genome sequencing using Illumina HiSeq2500. In total, 142.80 million PE reads for DF extreme bulks (66.34 million reads for EF and 76.46 million reads for late-flowering bulks, respectively) and 122.74 million PE reads (60.86 for obcordate leaf and 61.88 million reads for lanceolate leaf bulks, respectively) for leaf shape were generated. A total of 58.63 million PE reads were generated for ICP 5529 (Table 1). Alignment of the PE reads generated from ICP 5529 to the reference genome assembly of pigeonpea (Varshney et al. 2012) resulted in an average depth of 9.15X and 90.70% genome coverage, allowing us to develop a reference-guided assembly of ICP 5529 (hereafter designated as ICP 5529 assembly).

**Table 1:** Sequencing details of parental line and bulks and mapping of sequence reads.

Sample	Number of lines bulked	Total reads generated (Million reads)	High quality reads (Million reads)	Reads mapped (Million reads)	Genome coverage at 1X	Average depth (X)
ICP 5529^a^		58.63	35.33	15.33	90.70%	9.15
EF^b^	15	66.34	62.64	30.28	93.13%	15.10
LF^b^	15	76.46	63.99	30.25	93.26%	15.08
OLS^b^	15	60.86	51.13	26.86	92.93%	13.39
LLS^b^	15	61.88	52.23	26.90	92.95%	13.41

*EF* Early flowering pool, *LF* Late flowering pool, *OLS* Obcordate leaf shape pool, *LLS* Lanceolate leaf shape pool.

^a^ICP 5529 short reads were aligned to the publicly available pigeonpea genome of Asha (Varshney et al. 2012).

^b^The short reads of bulks were aligned to the ICP 5529 assembly developed by replacement of SNPs between ICP 5529 and Asha.

Mapping of the PE reads generated from extreme bulks to the developed ICP 5529 assembly for DF resulted in 15.08X and 15.10X sequencing depth and 93.26 and 93.13% coverage for late flowering (LF) and EF bulks, respectively. Similarly, for obcordate (OLS) and LLS bulks, we obtained alignment of 13.39X and 13.41X sequencing depth and 92.93 and 92.95% coverage, to the ICP 5529 assembly, respectively. The sequence alignment of LF bulk to the ICP 5529 assembly has provided 47,429 polymorphic SNPs. In this set, 9238 SNPs were homozygous in LF bulk. (Table S6). Similarly, of 46,510 identified SNPs between EF bulk and ICP 5529 assembly and 7427 were homozygous in EF bulk. In the case of leaf shape, 10,521 were homozygous from the total identified 54,556 SNPs for OLS bulk and 8770 were homozygous from total 51,658 SNPs for LLS bulk after mapping the bulk reads on to the ICP 5529 assembly (Table S7).

### Candidate genomic region(s) for days to flowering

**Fig. 1:**
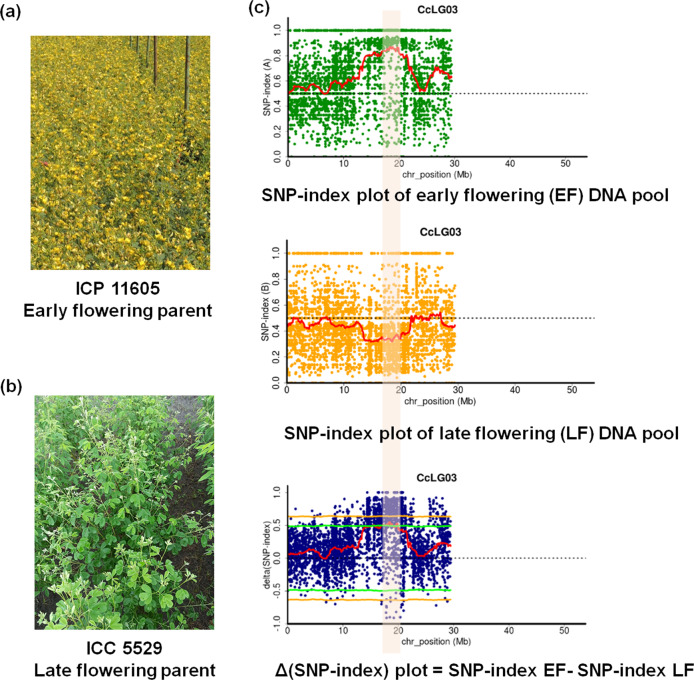
A QTL-seq approach to identify genomic regions controlling days to flowering in pigeonpea. **a** ICP 11605: early flowering parent; **b** ICP 5529: late flowering parent; **c** SNP index plot between early flowering pool (top), late flowering pool (middle) and ΔSNP index plot (bottom) of chromosome CcLG03 with statistical confidence interval under the null hypothesis of no QTLs (orange, *P* < 0.01; and green, *P* < 0.05). The significant genomic region identified for days to flowering is shaded (1.58 Mb region spanned through 19.22– 20.80 Mb).

QTL-seq analysis for DF revealed a genomic region on CcLG03 (Fig. 1, Table S8 and Figs S2–S4). A genomic region spanning 1.58 Mb (19.22–20.80 Mb) on CcLG03 showing significant (*P* < 0.05) deviation from equal inheritance of the two parental genomes had 56 SNPs with ∆SNP-index = −1 (Table S8). Of these 56 SNPs, nine SNPs were present in seven putative genes (Table 2). Of these nine genic SNPs, seven were in the intronic region and two SNPs were predicted in the exon regions of gene *C.cajan_09900* and *C.cajan_10078*, associated with pentatricopeptide repeat-containing protein and cell division protein, respectively. Substitution of SNPs in the predicted gene *C.cajan_09900* causing a non-synonymous substitution from Cysteine (aCg in ICP 5529 and LF pool) to Threonine (aTg in EF pool). Whereas, synonymous substitution in the predicted gene *C.cajan_10078* were observed between the pools.

**Table 2:** Identification of SNPs in putative candidate genes for days to flowering.

Linkage group	Gene	Position	ICP 5529 allele	LF allele	SNP index (LF pool)^a^	EF allele	SNP index (EF pool)^b^	Δ SNP-index^c^	SNP effect	Function
CcLG03	*C.cajan_09900*	19222701	G (aCg)^d^	G (aCg)^d^	0	A (aTg)^d^	1	−1	Exon (nsSNP)	Pentatricopeptide repeat-containing protein
CcLG03	*C.cajan_09938*	19549631	A	A	0	G	1	−1	Intron	Chromodomain-helicase-DNA-binding protein 4
CcLG03	*C.cajan_09958*	19690312	G	G	0	A	1	−1	Intron	Maestro heat-like repeat-containing protein family
CcLG03	*C.cajan_09965*	19763754	C	C	0	T	1	−1	Intron	Phosphatidylinositol 4-phosphate 5-kinase 9
CcLG03	*C.cajan_10046*	20439904	G	G	0	C	1	−1	Intron	1,4-alpha-glucan-branching enzyme
CcLG03	*C.cajan_10067*	20635496	T	T	0	A	1	−1	Intron	Uridine nucleosidase 1
CcLG03	*C.cajan_10078*	20745506	G	G	0	T	1	−1	Exon (sSNP)	Cell division protein
CcLG03	*C.cajan_10078*	20745771	A	A	0	G	1	−1	Intron	
CcLG03	*C.cajan_10078*	20747419	C	C	0	T	1	−1	Intron	

*nsSNP* non-synonymous SNPS, *sSNP* synonymous SNP.

^a^SNP-index of late flowering (LF) was calculated based on the allele calls and read depth in comparison to ICP 5529 reference assembly.

^b^SNP-index of early flowering (EF) bulk was calculated based on the allele calls and read depth in comparison to ICP 5529 reference assembly.

^c^Δ SNP-index of each SNP positions was calculated using following formula: Δ SNP-index = SNP-index of LF − SNP-index of EF.

^d^Value in parenthesis indicates the codon change due to SNP/Code for changed amino acids.

### Candidate genomic region(s) for leaf shape

**Fig. 2:**
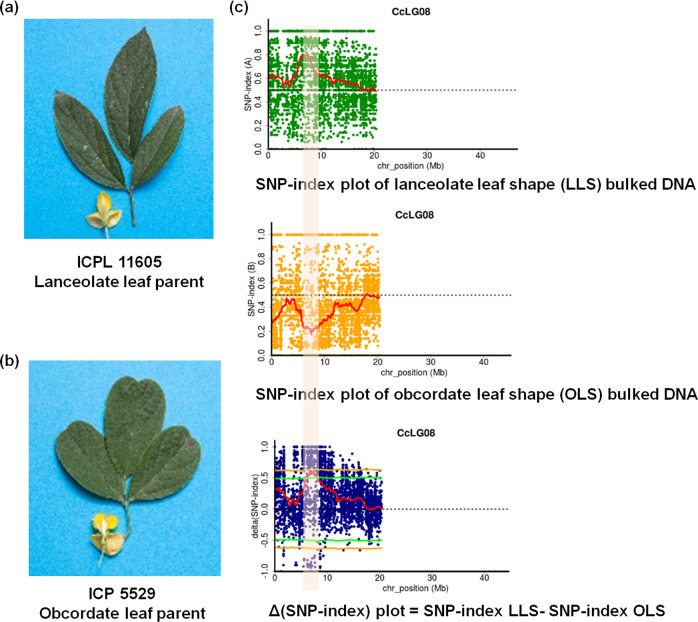
A QTL-seq approach to identify genomic regions controlling obcordate leaf shape in pigeonpea. **a** ICP 11605: parent of lanceolate type leaf; **b** ICP 5529: parent of obcordate type leaf; **c** SNP index plot between lanceolate leaf shape DNA pool (top), obcordate leaf shape DNA pool (middle) and ΔSNP index plot (bottom) of chromosome CcLG08 with statistical confidence interval under the null hypothesis of no QTLs (orange, *P* < 0.01; and green, *P* < 0.05). The significant genomic region identified for obcordate leaf shape is shaded (2.18 Mb region spanned from 6.69 to 8.88 Mb).

Sequence analysis of lanceolate and obcordate leaf bulks revealed a candidate genomic region on CcLG08 (Fig. 2, Table S9 and Figs. S5–S7). The genomic region spanning 2.19 Mb region (6.69–8.88 Mb) on CcLG08 revealed 210 SNPs with ∆SNP-index = −1, suggesting a biased inheritance of parental genomes in the two bulks (Table S9). The obcordate leaf bulk showed SNP-index = 0 indicating that obcordate alleles were inherited from the obcordate leaf parent ICP 5529. By contrast, lanceolate leaf bulk at these 210 positions possesses SNP-index = 1, indicating that their alleles were derived from the lanceolate leaf parent ICP 11605 (Table S9). Of the 210 SNPs, 39 SNPs were found in the genic regions of 20 genes. Of these 20 genic SNPs, 12 were present in the intronic region and eight SNPs were predicted in the exonic region of the genes (Table 3). SNP effect analysis of the eight exonic SNPs showed four synonymous and four non-synonymous substitutions. Substitution of SNPs in the predicted gene *C.cajan_15991* and *C.cajan_16002* causing a non-synonymous substitution from Threonine (Tgt/aTg in ICP 5529 and obcordate leaf pool) to Cysteine (Cgt/aCg in lanceolate leaf pool). Similarly, for two genes namely, *C.cajan_16012* [Glycine (Gca in ICP 5529 and obcordate leaf pool) to Alanine (Aca in lanceolate leaf pool)] and *C.cajan_16013* [Cysteine (cCt in ICP 5529 and obcordate leaf pool) to Threonine (cTt in lanceolate leaf pool) nsSNPs substitution was observed.

**Table 3:** Identification of SNPs in putative candidate genes for leaf shape.

Linkage group	Gene	Position	ICP 5529 allele	OLS allele	SNP index (OLS pool)^a^	LLS allele	SNP index (LLS Pool)^b^	Δ SNP-index^c^	SNP Effect	Function
CcLG08	*C.cajan_15985*	6701814	A	A	0	T	1	−1	Intron	Beta-carotene hydroxylase 2
CcLG08	*C.cajan_15991*	6764651	T (Tgt)^d^	T (Tgt)^d^	0	C (Cgt)^d^	1	−1	Exon (nsSNP)	Ac-like transposase
CcLG08	*C.cajan_16002*	6915910	T (aTg)^d^	T (aTg)^d^	0	C (aCg)^d^	1	−1	Exon (nsSNP)	Uncharacterized protein
CcLG08	*C.cajan_16003*	6921340	A	A	0	G	1	−1	Exon (sSNP)	Pro-Pol polyprotein
CcLG08	*C.cajan_16003*	6923929	T	T	0	C	1	−1	Intron	
CcLG08	*C.cajan_16003*	6924854	A	A	0	G	1	−1	Intron	
CcLG08	*C.cajan_16003*	6927480	C	C	0	T	1	−1	Intron	
CcLG08	*C.cajan_16003*	6927533	A	A	0	G	1	−1	Intron	
CcLG08	*C.cajan_16003*	6927560	T	T	0	C	1	−1	Intron	
CcLG08	*C.cajan_16003*	6927678	T	T	0	C	1	−1	Intron	
CcLG08	*C.cajan_16012*	7057478	G (Gca)^d^	G (Gca)^d^	0	A (Aca)^d^	1	−1	Exon (nsSNP)	F-box protein
CcLG08	*C.cajan_16012*	7059171	T	T	0	C	1	−1	Intron	
CcLG08	*C.cajan_16013*	7068488	G	G	0	C	1	−1	Intron	Uncharacterized protein
CcLG08	*C.cajan_16013*	7068679	G	G	0	A	1	−	Intron	
CcLG08	*C.cajan_16013*	7070780	C (cCt)^d^	C (cCt)^d^	0	T (cTt)^d^	1	−1	Exon (nsSNP)	
CcLG08	*C.cajan_16014*	7083922	T	T	0	A	1	−1	Intron	Transcriptional corepressor
CcLG08	*C.cajan_16014*	7093751	G	G	0	A	1	−1	Intron	
CcLG08	*C.cajan_16038*	7456634	T	T	0	C	1	−1	Intron	Cytochrome P450
CcLG08	*C.cajan_16038*	7456764	A	A	0	G	1	−1	Intron	
CcLG08	*C.cajan_16038*	7456831	T	T	0	A	1	−1	Intron	
CcLG08	*C.cajan_16038*	7456974	A	A	0	G	1	−1	Intron	
CcLG08	*C.cajan_16038*	7457844	C	C	0	A	1	−1	Intron	
CcLG08	*C.cajan_16041*	7486941	A	A	0	G	1	−1	Intron	Uncharacterized protein
CcLG08	*C.cajan_16047*	7606346	T	T	0	A	1	−1	Intron	Transposon Ty3-I
CcLG08	*C.cajan_16049*	7641790	A	A	0	C	1	−1	Intron	E3 ubiquitin-protein ligase
CcLG08	*C.cajan_16049*	7642733	G	G	0	A	1	−1	Intron	
CcLG08	*C.cajan_16049*	7643315	A	A	0	C	1	−1	Exon (sSNP)	
CcLG08	*C.cajan_16051*	7666784	A	A	0	G	1	−1	Intron	Protein ROOT PRIMORDIUM DEFECTIVE 1
CcLG08	*C.cajan_16051*	7667174	T	T	0	C	1	−1	Intron	
CcLG08	*C.cajan_16059*	7780600	A	A	0	T	1	−1	Intron	Probable methyltransferase PMT16
CcLG08	*C.cajan_16061*	7838639	C	C	0	T	1	−1	Exon (sSNP)	1-aminocyclopropane-1-carboxylate oxidase homolog 1
CcLG08	*C.cajan_16062*	7870949	A	A	0	G	1	−1	Intron	
CcLG08	*C.cajan_16063*	7888623	G	G	0	A	1	−1	Intron	1-aminocyclopropane-1-carboxylate oxidase homolog 12
CcLG08	*C.cajan_16066*	7940765	C	C	0	G	1	−1	Intron	Tripeptidyl-peptidase 2
CcLG08	*C.cajan_16066*	7947009	G	G	0	A	1	−1	Intron	
CcLG08	*C.cajan_16066*	7950673	A	A	0	C	1	−1	Intron	
CcLG08	*C.cajan_16068*	8008253	C	C	0	A	1	−1	Intron	Cytochrome P450
CcLG08	*C.cajan_16074*	8168767	C	C	0	A	1	−1	Intron	Type I inositol
CcLG08	*C.cajan_16099*	8666995	T	T	0	C	1	−1	Exon (sSNP)	-

*nsSNP* non-synonymous SNPS, *sSNP* synonymous SN.

^a^SNP-index of obcordate leaf shape (OLS) bulk was calculated based on the allele calls and read depth in comparison to ICP 5529 reference assembly.

^b^SNP-index of lanceolate leaf shape (LLS) bulk was calculated based on the allele calls and read depth in comparison to ICP 5529 reference assembly.

^c^Δ SNP-index of each SNP positions was calculated using following formula: Δ SNP-index = SNP-index of OLS − SNP-index of LLS.

^d^Value in parenthesis indicates the codon change due to SNP/Code for changed amino acids.

## Discussion

Advances in genomics have led to the development of various NGS based rapid trait mapping approaches like QTL-seq, MutMap, Indel-seq, BSA-Seq, etc (Varshney et al. 2020). NGS technologies have enabled modification and improvement of traditionally tricky, time-consuming bulked segregant analysis (BSA, Michelmore et al. 1991) into rapid and whole genome sequencing-based high-resolution trait mapping (Schlötterer et al. 2014). This approach has become popular nowadays due to affordable sequencing cost to many research groups and high throughput NGS tools. Moreover, the availability of draft genome sequence information in a species speeds up the sequencing of multiple individuals of that species and allows rapid identification of genomic variations as well as mapping and isolation of genes for causative mutations/target traits. Sequencing-based trait mapping combines both classical genetics and NGS platforms to map the associated traits. The application of sequencing-based trait mapping can be divided into two classes (i) trait mapping through bulk sequencing of populations, and (ii) trait mapping through complete sequencing of populations. Several examples of NGS-based trait mapping have been reported in many crop species (see Varshney et al. 2019).

### Genomic regions for leaf shape and days to flowering

Genome sequencing of pigeonpea opened new avenues to enable sequencing-based trait mapping (Varshney et al. 2012). Sequencing-based bulked segregant analysis combined with nsSNPs substitution-based approach were utilized to map the candidate genes for FW and SMD resistance in pigeonpea (Singh et al. 2016a). Similar to SNP-based approach, Indel-seq approach (Indels based) was proposed and utilized for the first time toward the identification of candidate genomic regions/genes for FW and SMD resistance in pigeonpea (Singh et al. 2017b). In the present study, the sequencing of both bulks (EF and LF bulks) identified a comparatively large number of homozygous SNPs (9238 and 7427 SNPs for LF and EF, respectively). The identified SNPs were utilized to calculate the genome-wide SNP index information for both the pools. Analysis of SNPs located in the mapped candidate region on CcLG03 revealed a candidate gene, *C.cajan_09900* coding for pentatricopeptide repeat (PPR) containing protein. It is well documented in the literature that PPR protein regulates flowering time in Arabidopsis (Emami et al. 2019). The role of *C.cajan_09900* in the EF of pigeonpea can now be investigated further. We have also identified SNPs in the exonic regions of the gene *C.cajan_10078*, which codes for the cell division protein *FtsZ* homolog, however, a specific role of this gene in flowering not been reported. These two candidate genes especially gene *C.cajan_09900* with non-synonymous mutation and few other genes discovered earlier in pigeonpea using candidate gene approach namely *CcTFL1* and *EARLY FLOWERING3* (Saxena et al. 2017c; Varshney et al. 2017) would be useful in expanding our understanding of molecular mechanism involved in flowering in pigeonpea and also in related legume species. Furthermore, sequence variations detected in these genes will facilitate the development of EF cultivars in pigeonpea through genomics-assisted breeding (Varshney et al. 2021). Flowering time in pigeonpea is critical as it directly correlates with the maturity of the plant. Nowadays as research efforts are being directed toward development of short duration or EF and early maturing pigeonpea varieties that mature around 100–120 days or early, the present findings will facilitate crop improvement programs. The short duration pigeonpea varieties will provide opportunities to include them in the existing cereal based cropping systems and expand to new niches where pigeonpea could not be cultivated due to their LF or late maturity and photo-sensitivity.

Similarly, for obcordate leaf shape, a genomic region on CaLG08 was identified with four non-synonymous SNPs in four different genes (*C.cajan_15991, C.cajan_16002, C.cajan_16012* and *C.cajan_16013*). Identified candidate gene *C.cajan_15991* coding for Ac-like transposase has been earlier reported to play an important role in wrinkled shape character in pea (Bhattacharyya et al. 1990) and thus more understanding of this gene will be required to prove any possible role in the leaf shape of pigeonpea. We have also identified candidate gene *C.cajan_16012*, which codes for F-box protein. Previously, the role of F-box protein has been identified in leaf size and shape (Baute et al. 2017). Two identified candidate genes *C.cajan_16002* and *C.cajan_16013* have been reported as uncharacterized protein, thus more understanding and functional characterization will be required to understand their role in leaf shape development. These results highlighted the significance of the QTL-seq approach in identifying refined and reliable candidate regions for the traits of interest.

### Co-localization of genomic regions with QTLs identified through genetic linkage mapping approach

Further, to validate our results obtained through QTL-seq approach, we have used available genetic map information on ICP 5529 × ICP 11605 population (Table S10) (Obala et al. 2019). The phenotyping data obtained on entire population for DF were combined with the genetic map information to perform classical QTL analysis. Composite interval mapping identified a total of four QTLs for DF on CcLG03 with PVE ranging from 4.60 to 47.58% (Table S11). Three QTLs, namely *qDF3.1* (39.58%), *qDF3.3* (47.58%) and *qDF3.4* (16.18%) were identified as major effect QTLs and remaining one QTL showed minor effects (*qDF3.2*) for DF. All the four identified QTLs for DF were mapped between 16.68 and 22.23 Mb (5.55 Mb) region on CcLG03. We have also identified the number of genes present within each QTL region and a minimum of 32 genes were identified within QTL, *qDF3.3*, while a maximum of 588 genes were identified within QTL, *qDF3.1*. All the four QTLs were found in these regions with different spans of the QTL window. Therefore, it is difficult to select the genes/genomic regions for molecular breeding without narrowing the candidate genomic regions. Interestingly, the QTL-seq approach also identified the DF related QTLs with a much narrow window (1.58 Mb region; 19.22–20.80 Mb) of the QTLs on CaLG03 (Table S12). Comparative to 588 coding genes reported in the conventional QTL approach, QTL-seq provided the opportunity to select the candidate genes from the identified two exonic SNPs. After validation of these identified exonic SNPs, these can be utilized in the crop improvement programs for the development of early maturing pigeonpea varieties.

### Comparative genetic mapping of days to flowering related genes across key legume crops

To utilize the identified genomic regions in pigeonpea associated with DF, we tried to understand the relevance of the present study in other crops of *Fabaceae* family. We did a comparative mapping of the identified seven *Cajanus cajan* genes associated with DF with 12 crop genomes (*(i) Arachis duranensis (Aradu), (ii) Arachis hypogaea (Arahy), (iii) Arachis ipaensis (Araip), (iv) Cicer arietinum, (v) Glycine max (Glyma), (vi) Glycine soja (Glyso), (vii) Lupinus angustifolius (Lupan), (viii) Medicago truncatula (Medtr), (ix) Phaseolus vulgaris (Phavu), (x) Vigna angularis (Vigan), (xi), Vigna radiata (Vigra) (xii) Vigna unguiculata (Vigun)*.) (Fig. S8). We have performed protein blast using DELTA-BLASTP. Query coverage >90%, similarity >60% and e-value ≤ 1e^−10^ were taken into consideration for best predicted results. Protein domain and gene functional analysis were performed using Interpro (Table S13). We have found two genes, earlier reported which plays an important role in flowering in Arabidopsis. The interpro domain prediction provided similar domain region hits in the protein structure of PPR domain *C.cajan_09900* reflected Pentatricopeptide repeat in its protein structure. PRECOCIOUS1 (*POCO1*), a p-class PPR repeat protein reported as to affect flowering time in *Arabidopsis thaliana* (Emami et al. 2019). Another gene *C.cajan_09938* hits the Zinc finger PHD-type protein named PHD finger domain containing protein (PFD) is identified to suppress the flowering in *Arabidopsis thaliana* (Yokoyama et al. 2019). Conserved genomic regions were identified with nine genomes out of the selected 12 targeted genomes. A high degree of conserved collinear synteny among the chromosome 6 of *Aradu, Arahy and Araip* were identified. On chromosome 3 of *Cicer*, chromosome 19 of *Glyma* and *Glyso* and chromosome 3, 1 and 7 of *Vigra*, *Phavu* and *Medtr* respectively, we have also found the similarity with the identified *Cajanus cajan* genes (Table S14). An interpretation in the view of domain search of candidate genes can give proportional understanding of functions. Comparative analysis of the seven *Cajanus cajan* genes with the 12 genomes, mapped to 153 homologous genes ranged from 13 (*C.cajan_09900* and *C.cajan_09958*) to 36 (*C.cajan_09965*). The number of genes mapped to the other crop genomes ranged from 9 (*Cicer areitinum* and *Vigna radiata*) to 20 (*Glycine max*) (Table S15). Identified genes in the present study through synteny analysis in the other crops can be validated and utilized for the development of early duration crop varieties.

## Conclusion

The present study has provided candidate genes for DF and leaf shape in pigeonpea using QTL-seq approach. Some candidate genes have been prioritized using information on non-synonymous SNPs and homology based analysis. Moreover, we have also enriched the genomic regions associated with DF through GBS based QTL analysis. In summary, this study has provided the most promising candidate gene/s for DF (*C.cajan_09900*) and leaf shape (*C.cajan_15991, C.cajan_16002, C.cajan_16012* and *C.cajan_16013*) in pigeonpea. Additionally, comparative genetic mapping of DF related genes with other crops, revealed several important genes in nine other *Fabaceae* crop genomes. These candidate genes will be helpful in identification, cloning and functional validation of causal mutation or gene/s responsible for DF and leaf shape in pigeonpea and other related legume species.

## Supplementary information

Supplemental information

Supplemental information

## Data Availability

All the data generated in the present study is provided in the Supplementary Information and sequencing data deposited as Bioproject ID PRJNA774652 in NCBI.
