# Supplementary material for: QTL-seq for the identification of candidate genes for days to flowering and leaf shape in pigeonpea
Source: Heredity (Edinb). 2022 Jan 12;128(6):411–9. doi: 10.1038/s41437-021-00486-x (PMC9177671; doi:10.1038/s41437-021-00486-x)
Supplement: Supplementary file 1 — Supplemental information [file 41437_2021_486_MOESM1_ESM.docx]

**Supplementary information**

**QTL-seq for the identification of candidate genes for days to flowering and leaf shape in pigeonpea**

**Vikas Singh^1,2,#^, Pallavi Sinha^1,2,#^, Jimmy Obala^1, 3, #^, Aamir W Khan^1^,**

**Annapurna Chitikineni^1^, Rachit K Saxena^1^, Rajeev K Varshney^1,4,^** **^*^**

^1^Center of Excellence in Genomics & Systems Biology, International Crops Research Institute for the Semi-Arid Tropics (ICRISAT), Hyderabad 502 324, India

^2^International Rice Research Institute (IRRI), South-Asia Hub, ICRISAT, Hyderabad, India.

^3^Department of Science, Lira University, Lira City, Uganda

^4^State Agricultural Biotechnology Centre, Centre for Crop and Food Innovation, Food Futures Institute, Murdoch University, Murdoch WA6150, Western Australia, Australia

**^*^Author for Correspondence**

Rajeev K Varshney

International Crops Research Institute for the Semi-Arid Tropics (ICRISAT)

Patancheru - 502 324, India

Telephone: 91-40-30713305;

Fax: 91-40-30713074

Email: [r.k.varshney@cgiar.org](mailto:r.k.varshney@cgiar.org);

**
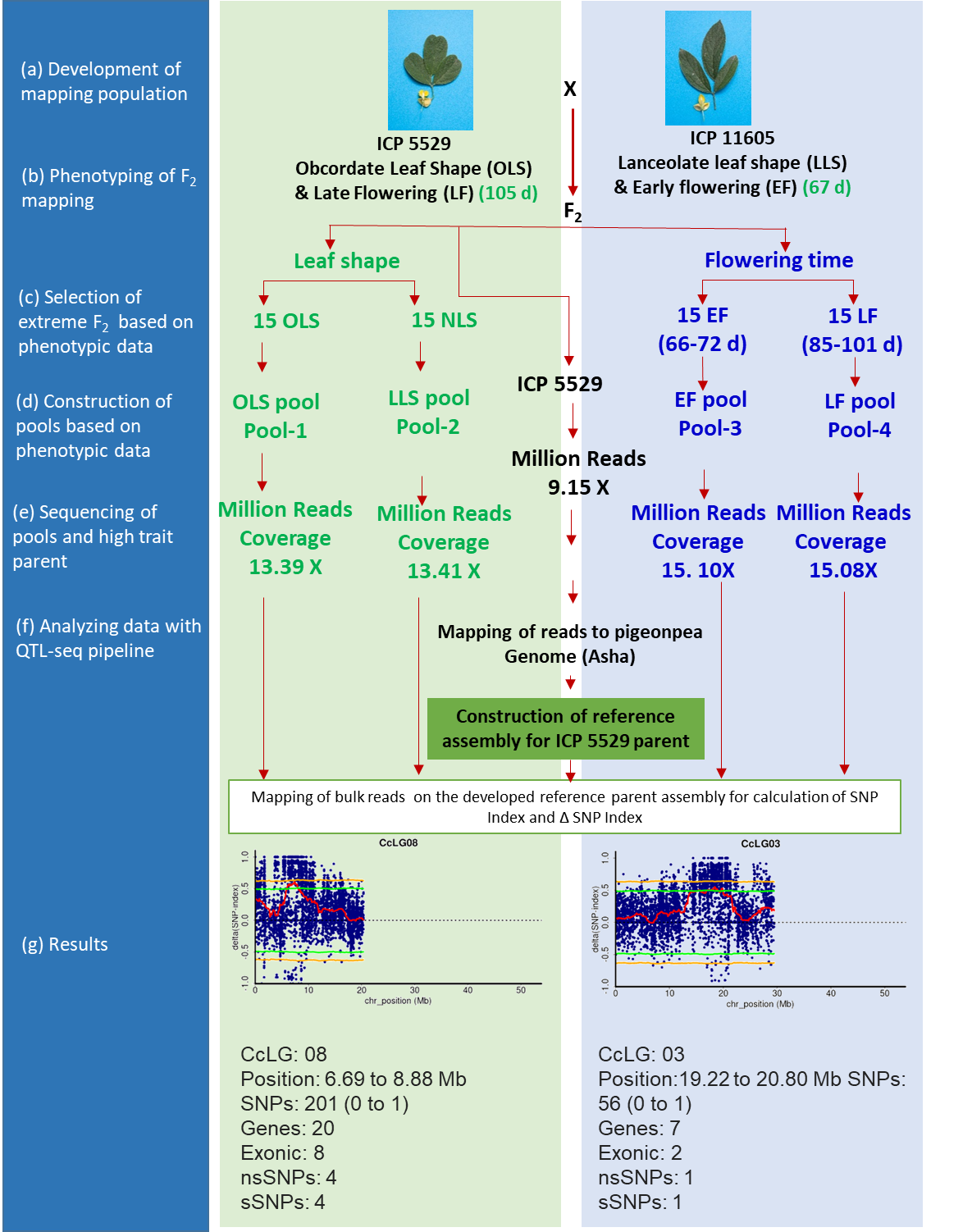
**

**Figure S1.** **QTL-seq approach used in pigeonpea for leaf shape and days to flowering** (a) Two contrasting parents namely ICP 5529 (obcordate leaf shape; OLS and late flowering; LF) and ICP 11605 (lanceolate leaf shape; LLS and early flowering, EF) were crossed to develop F_2_ population. (b) Developed F_2_ were phenotyped for targeted traits (leaf shape and flowering duration) (c) Two sets of F_2_ pools were selected for constructed of days to flowering pools (early flowering, EF pool and late flowering, LF pool) and leaf shape pools (obcordate leaf shape pool-OLS and lanceolate leaf shape pool-LLS) by pooling 15 plants in each of the class (d) Two sets of DNA bulks were constituted based on equimolar mixing of DNAs of individuals. (e) These two sets of DNA bulks along with the OLS parent (ICP 5529) were applied to whole-genome re-sequencing for further identification of SNPs for calculation of SNP-index through QTL-seq pipeline. (f) The QTL-seq pipeline was used for identification of candidate genomic regions for both the traits. (g) Based on the ΔSNP-index values for individual bulks putatively linked candidate genomic regions/SNPs were identified.


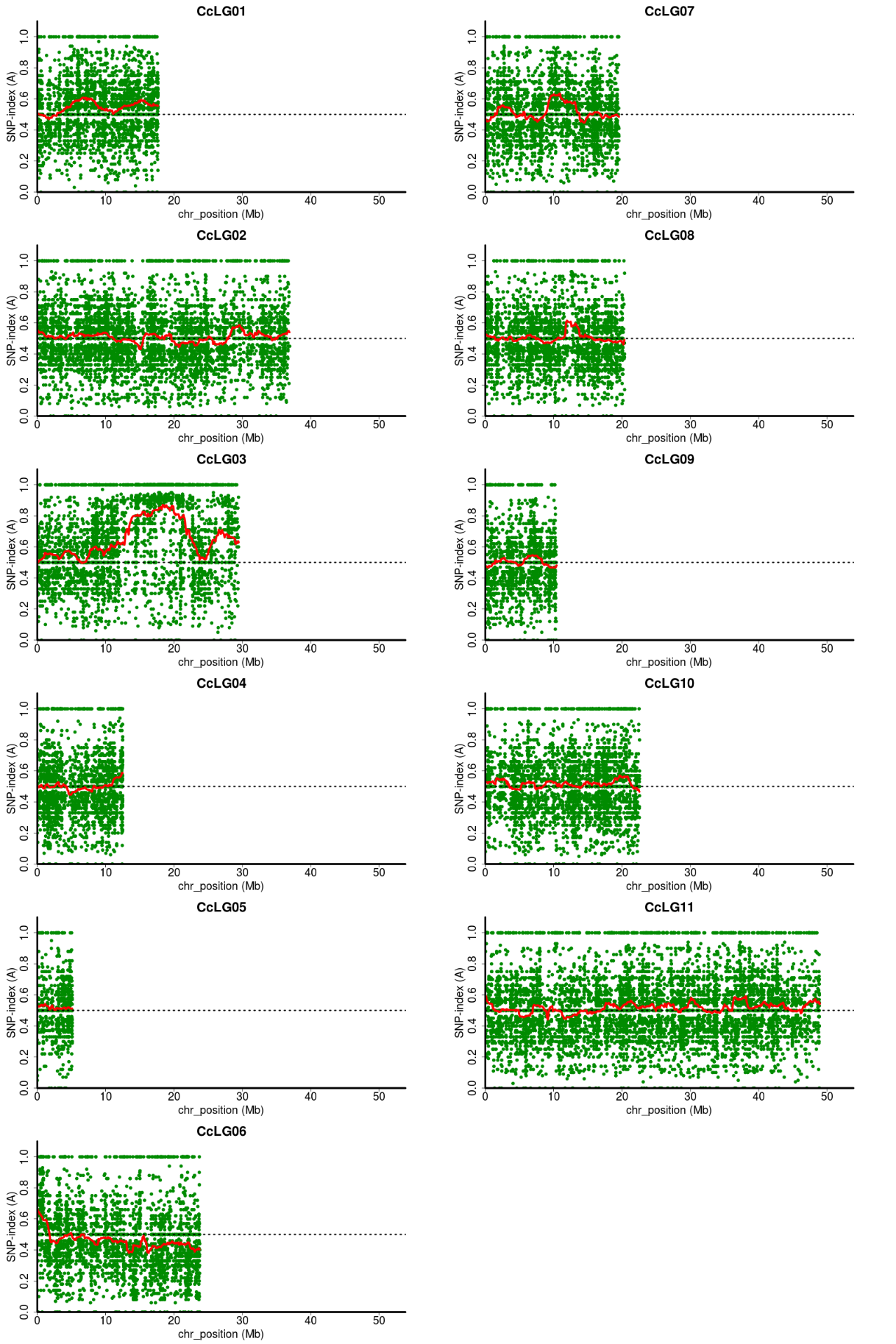


**Figure S2.** SNP-index plots for 11 chromosomes of early flowering (EF) bulked DNA. Red lines indicate the sliding window average of 2 Mb interval with 10 kb increment for SNP-index.


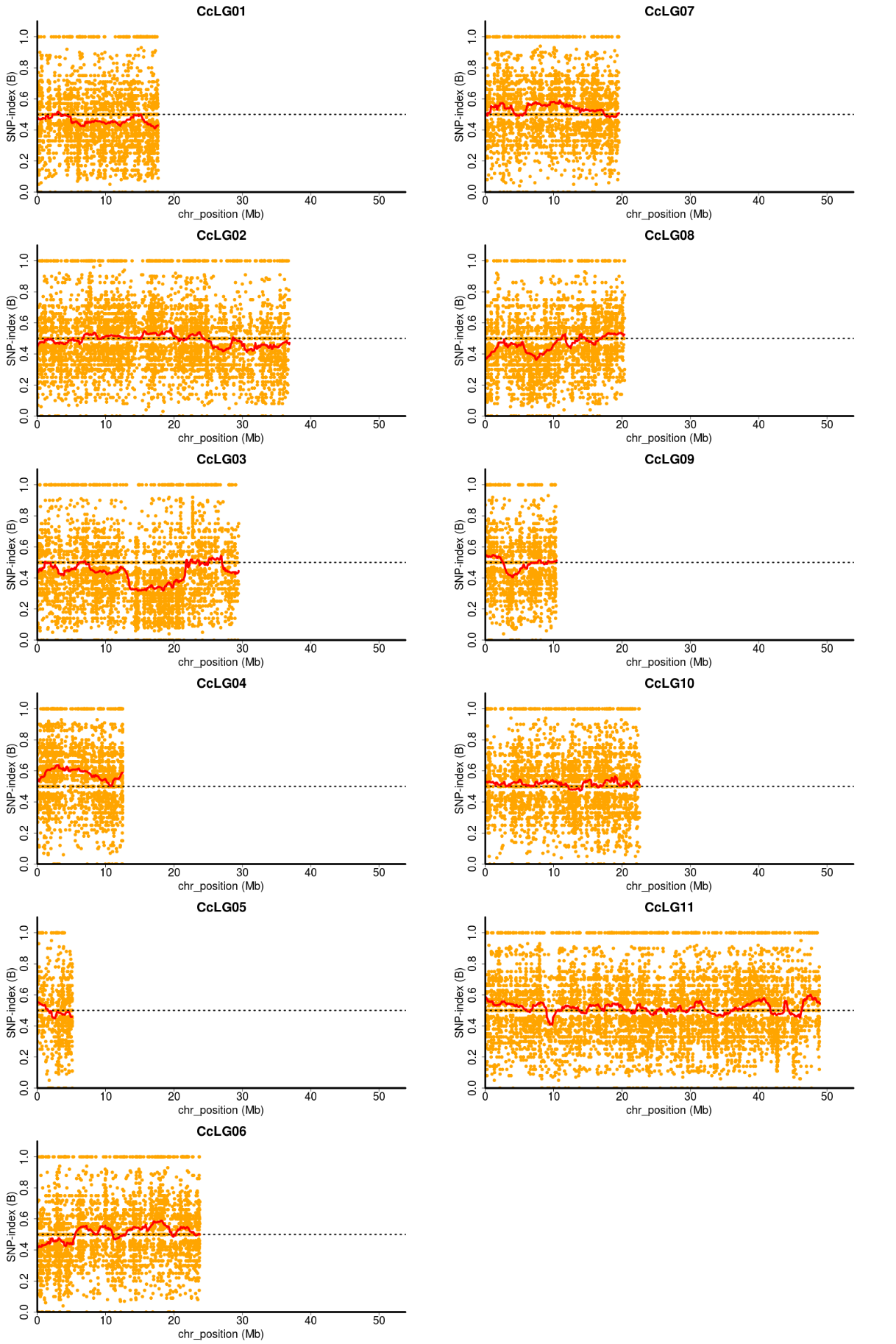


**Figure S3.** SNP-index plots for 11 chromosomes of late flowering (LF) bulked DNA. Red lines indicate the sliding window average of 2 Mb interval with 10 kb increment for SNP-index.


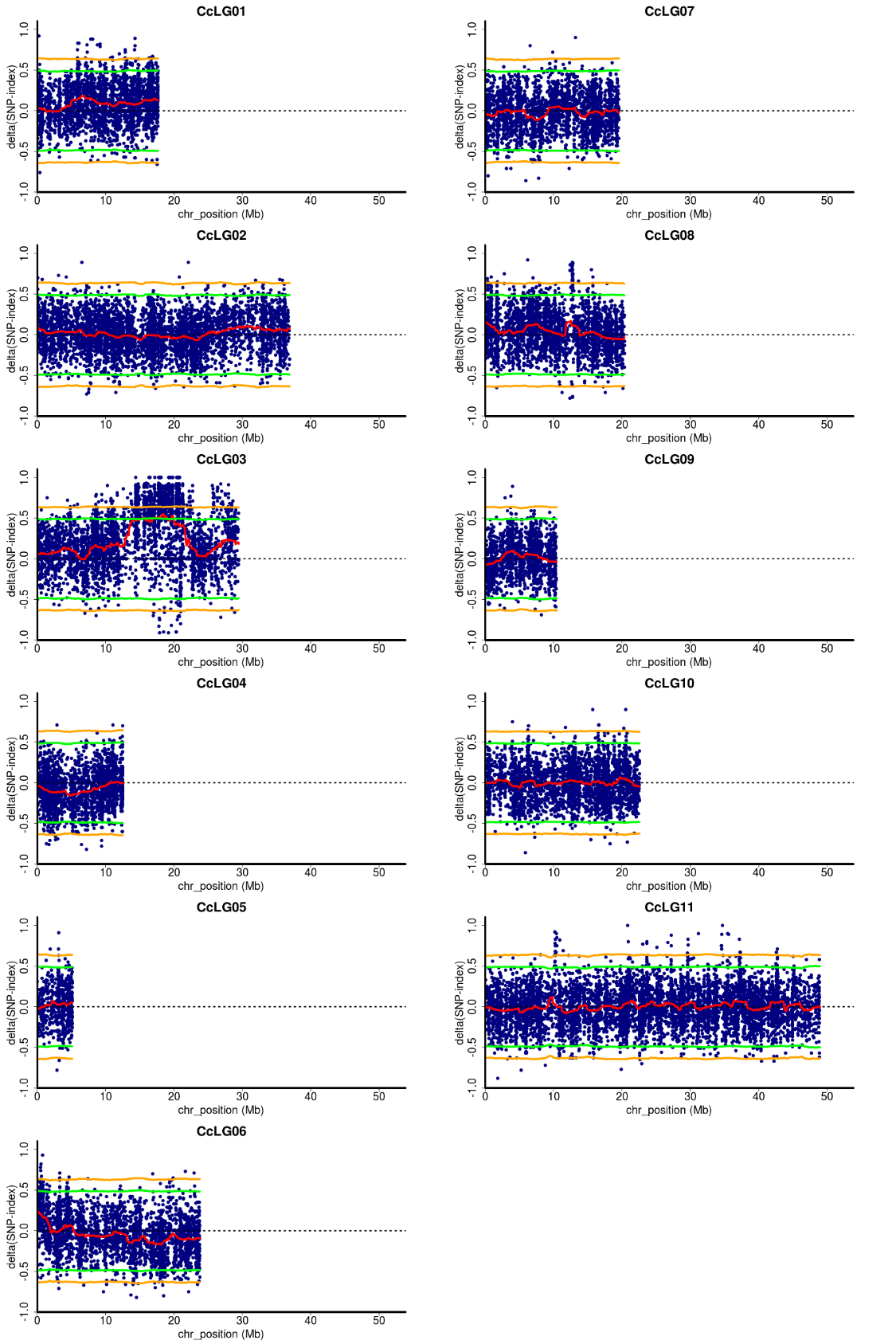


**Figure S4.** The Δ(SNP-index) plot obtained by subtraction of late flowering bulk SNP-index from early flowering bulk SNP-index for F_2_ obtained from a cross between ICP 5529 and ICP 11605. Statistical confidence intervals under the null hypothesis of no QTL are shown (green: P < 0.05; orange: P < 0.01).


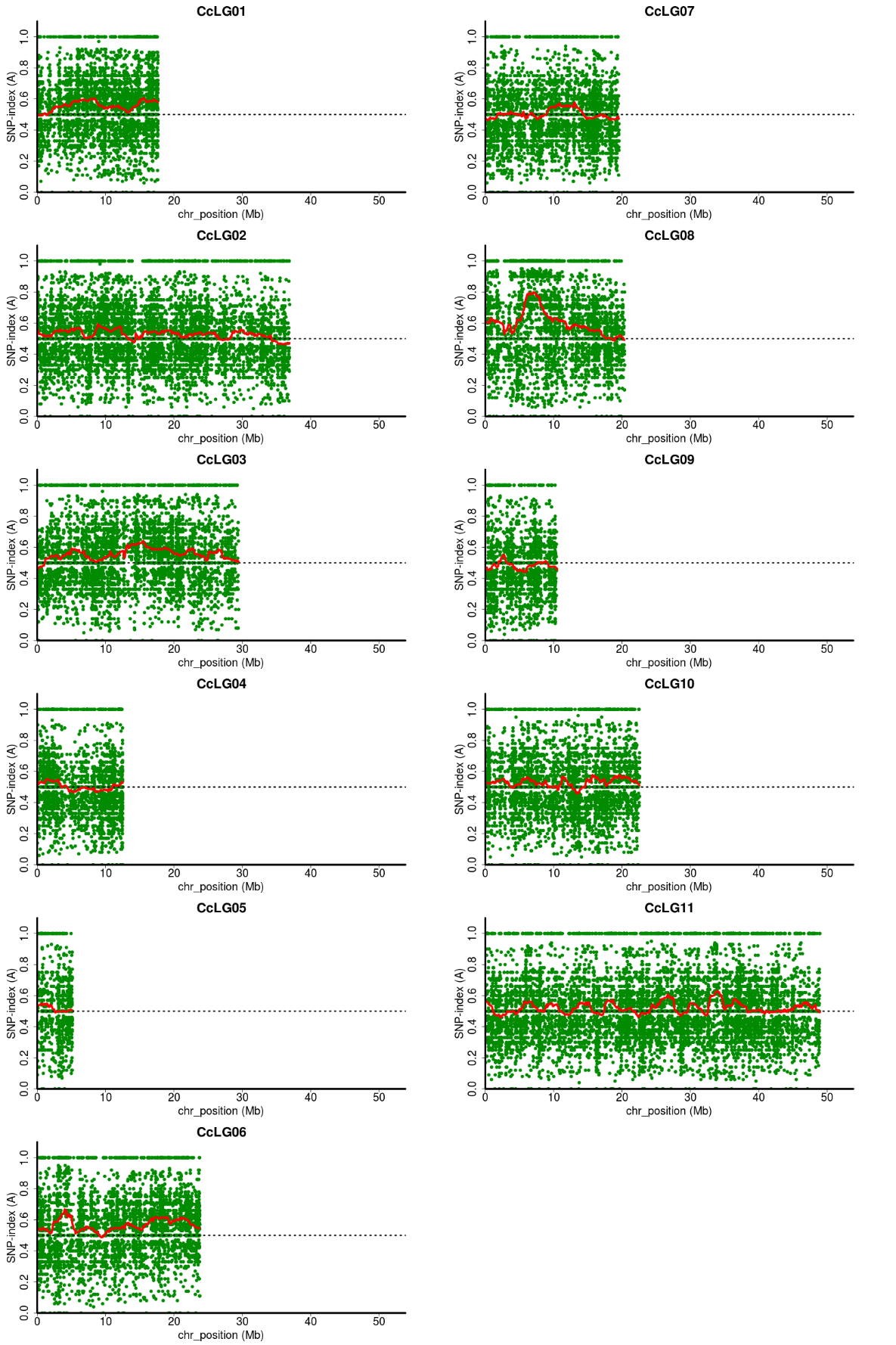


**Figure S5.** SNP-index plots for 11 chromosomes of obcordate leaf shape (OLS) bulked DNA. Red lines indicate the sliding window average of the 2 Mb interval with 10 kb increment for SNP-index.


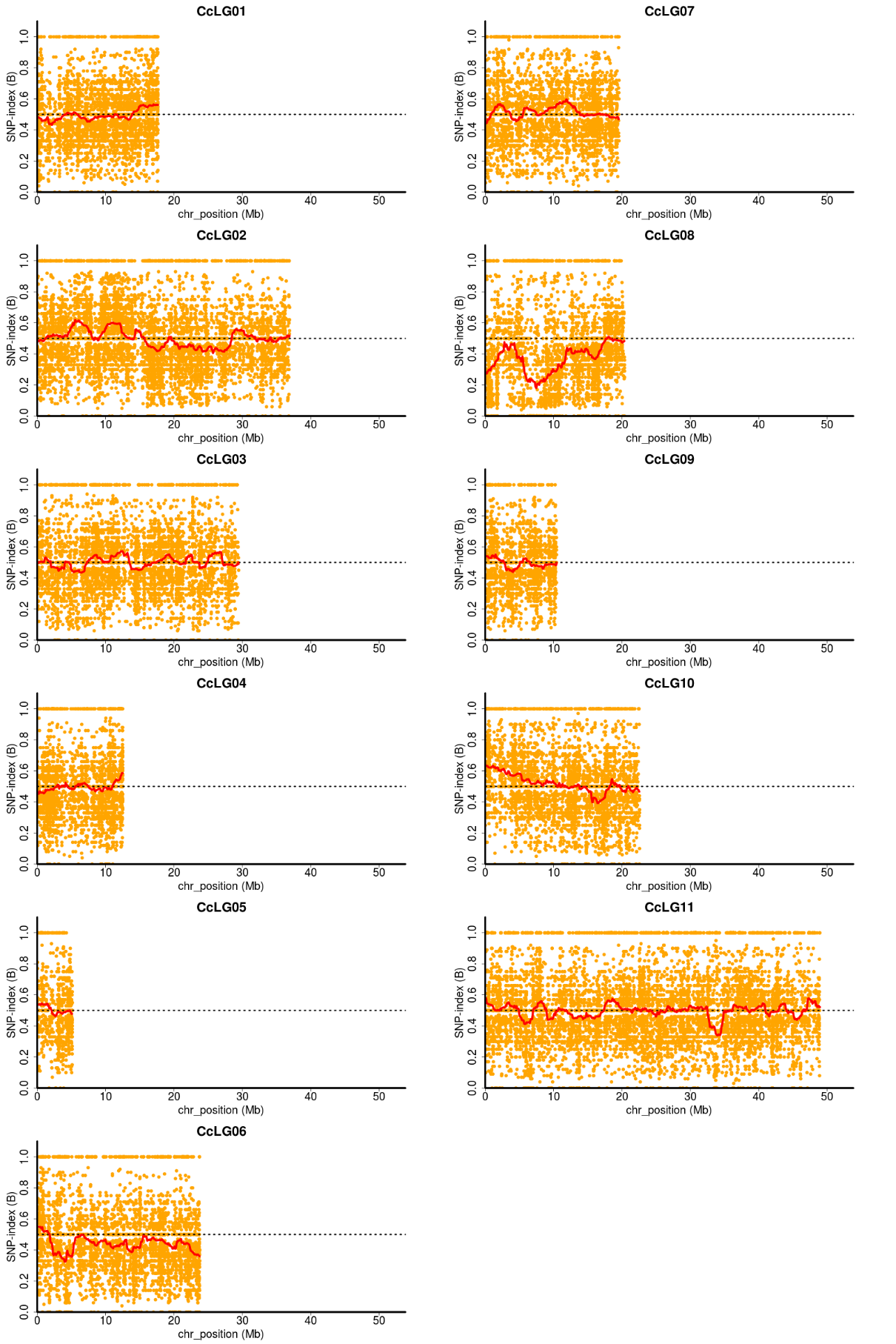


**Figure S6**. SNP-index plots for 11 chromosomes of lanceolate leaf shape (LLS) bulked DNA. Red lines indicate the sliding window average of 2 Mb interval with 10 kb increment for SNP-index.


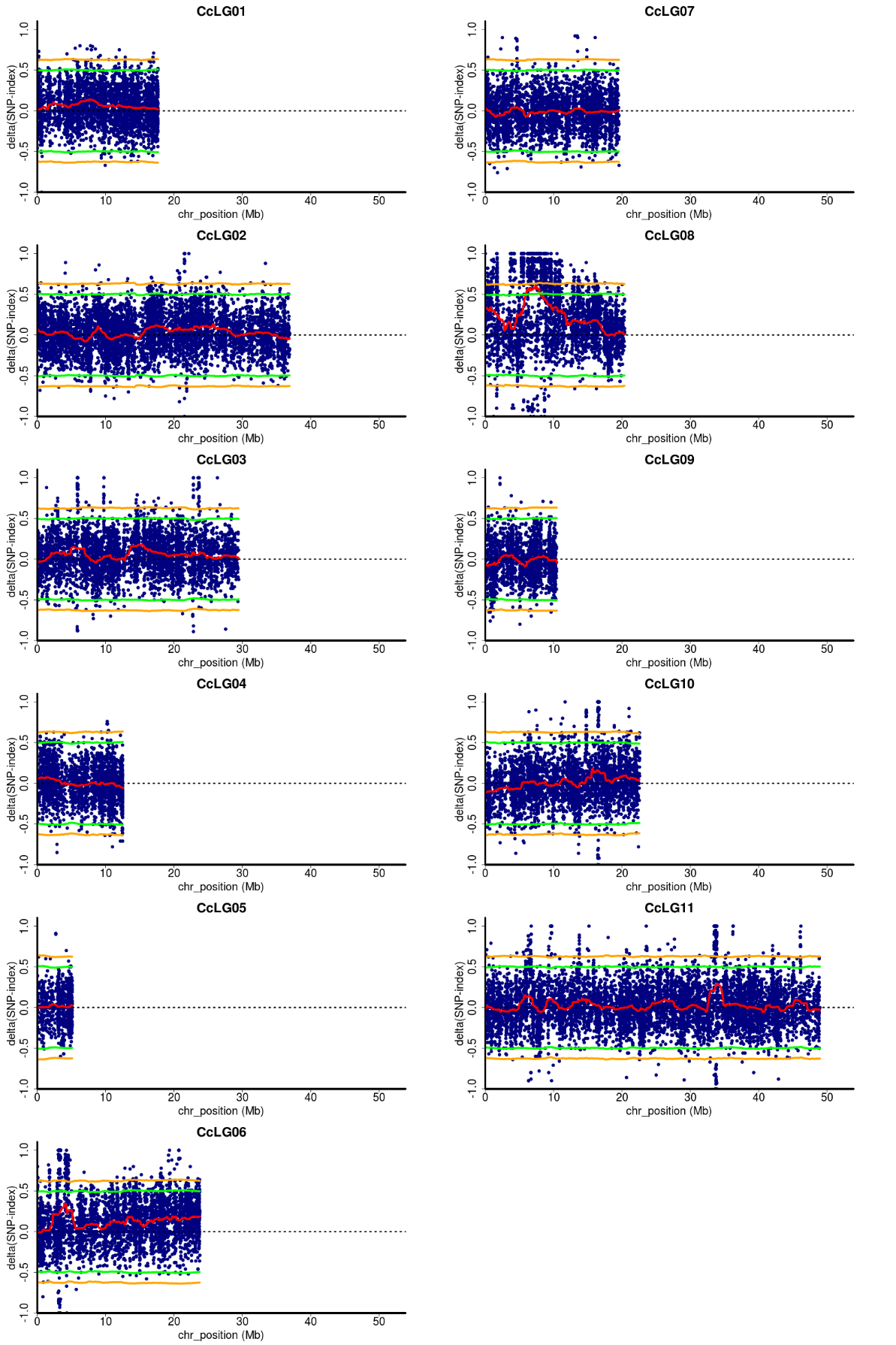


**Figure S7.** The Δ(SNP-index) plot obtained by subtraction of obcordate leaf shape SNP-index from lanceolate leaf shape SNP-index for F_2_ obtained from a cross between ICP 5529 and ICP 11605. Statistical confidence intervals under the null hypothesis of no QTL are shown (green: *P* < 0.05; orange: *P* < 0.01).


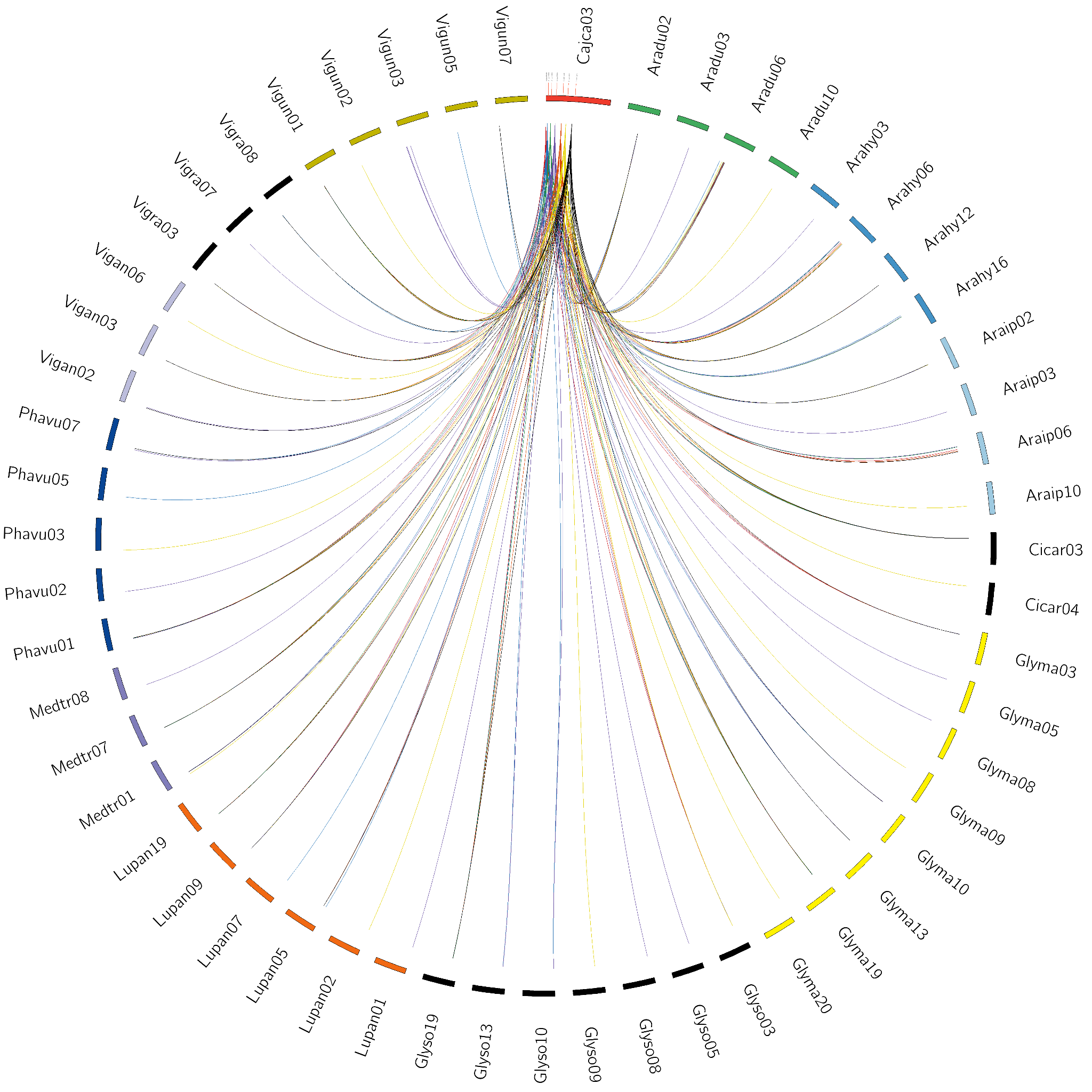


**Figure S8.** Comparative gene mapping of seven candidate genes identified in the genomic region associated with days to 50% flowering mapped on twelve *Fabaceae* genomes *(i) Arachis duranensis (Aradu), (ii) Arachis hypogea (Arahy), (iii) Arachis ipaensis (Araip), (iv) Cicer arietinum, (v) Glycine Max (Glyma), (vi) Glycine soja (Glyso), (vii) Lupinus angustifolius (Lupan), (viii) Medicago truncatula (Medtr), (ix) Phaseolus vulgaris (Phavu), (x) Vigna angularis (Vigan), (xi), Vigna radiata (Vigra) (xii) Vigna unguiculata (Vigun).* Circos graph depicted conserved syntenic genetic between the genes and their relationships among eleven legume genomes, which are depicted in the Circos circular ideogram. A high degree of conserved collinear synteny were mapped in 9 genomes. Among the chromosomes of 6 in *Aradu, Arahy* and *Araip*. Chromosome 3 of *Cicar*, chromosome 19 of *Glyma* and *Glyso* and chromosome 3, 1 ,7 of *Vigra, Phavu* and *Medtr* respectively.

**Table S1.** Phenotyping of F_2_ mapping population for days to flowering and leaf shape (attached as dataset in excel format)

Table S2 Descriptive statistics of F_2_ mapping population for days to flowering

| **Trait** | **ICP 5529** | **ICP 11605** | **\|ICP 5529-ICP 11605\|** | **F2** | **Mean ± s.d.** | **CV (%)** | **S** | **K** | **F_2_-range** | **W-test** |
| --- | --- | --- | --- | --- | --- | --- | --- | --- | --- | --- |
| DFF (days) | 104.0 | 66.0 | 38.0 | 179 | 81.2±9.2 | 11.3 | -0.2 | -0.5 | 65.0-102.0 | 0.9*** |

*, ** and ***: significantly different from a Gaussian distribution at 0.05, 0.01 and 0.001 probability levels, respectively. |ICP 5529-ICP 11605|: absolute difference in trait value between two parents of a cross, CV; coefficient of variation S: skewness, K: kurtosis

Table S3 Phenotype of leaf in F_1_ and segregation for leaf type in F_2_ generation

| **Cross** | **Phenotype of F_1_** | **Total # of F_2_ plants** | **Lanceolate Leaf** | **Obcordate leaf** | **X^2^ (3:1)** | **p-value** |
| --- | --- | --- | --- | --- | --- | --- |
| ICP 5529 | Obcordate leaf |  |  |  |  |  |
| ICP 11605 | Lanceolate leaf |  |  |  |  |  |
| ICP 5529 x ICP 11605 | Lanceolate leaf | 179 | 115 | 64 | 11.04 | 0.0009 |

**Table S4.** Summary of selected F_2_ lines to construct early (EF) and late flowering (LF) pools

| Early flowering pool (EF) | Days to flowering (days) | Late flowering pool (LF) | Days to flowering (days) |
| --- | --- | --- | --- |
| *Parents* |  | *Parents* |  |
| ICP 5529^ǂ^ | 105 | ICP 11605^ǂ^ | 67 |
| *ID of selected extreme F_2_* | | *ID of selected extreme F_2_* | |
| F2-ID-11 | 65 | F2-ID-6 | 102 |
| F2-ID-110 | 65 | F2-ID-46 | 102 |
| F2-ID-120 | 65 | F2-ID-71 | 101 |
| F2-ID-124 | 65 | F2-ID-80 | 101 |
| F2-ID-15 | 66 | F2-ID-108 | 100 |
| F2-ID-49 | 66 | F2-ID-131 | 98 |
| F2-ID-51 | 66 | F2-ID-8 | 96 |
| F2-ID-58 | 66 | F2-ID-75 | 96 |
| F2-ID-62 | 66 | F2-ID-103 | 96 |
| F2-ID-63 | 66 | F2-ID-60 | 95 |
| F2-ID-65 | 66 | F2-ID-115 | 94 |
| F2-ID-67 | 66 | F2-ID-123 | 93 |
| F2-ID-68 | 66 | F2-ID-153 | 93 |
| F2-ID-73 | 66 | F2-ID-119 | 92 |
| F2-ID-74 | 66 | F2-ID-143 | 92 |
| Pool Mean | 65.8 | Pool Mean | 95.4 |

^ǂ^Parents of F_2_ mapping population

**Table S5.** Summary of selected F_2_ lines to construct obcordate (OLS) and lanceolate (LLS) leaf shape pools

| OLS pools | Leaf shape type | LLS pools | Leaf shape type |
| --- | --- | --- | --- |
| *Parents* |  | *Parents* |  |
| ICP 5529^ǂ^ | Obcordate Leaf | ICP 11605^ǂ^ | Lanceolate Leaf |
| *ID of selected extreme F_2_* | | *ID of selected extreme F2* | |
| F2-ID-6 | Obcordate Leaf | F2-ID-46 | Lanceolate Leaf |
| F2-ID-71 | Obcordate Leaf | F2-ID-108 | Lanceolate Leaf |
| F2-ID-80 | Obcordate Leaf | F2-ID-131 | Lanceolate Leaf |
| F2-ID-75 | Obcordate Leaf | F2-ID-8 | Lanceolate Leaf |
| F2-ID-119 | Obcordate Leaf | F2-ID-103 | Lanceolate Leaf |
| F2-ID-143 | Obcordate Leaf | F2-ID-60 | Lanceolate Leaf |
| F2-ID-152 | Obcordate Leaf | F2-ID-115 | Lanceolate Leaf |
| F2-ID-135 | Obcordate Leaf | F2-ID-123 | Lanceolate Leaf |
| F2-ID-111 | Obcordate Leaf | F2-ID-153 | Lanceolate Leaf |
| F2-ID-3 | Obcordate Leaf | F2-ID-145 | Lanceolate Leaf |
| F2-ID-27 | Obcordate Leaf | F2-ID-147 | Lanceolate Leaf |
| F2-ID-36 | Obcordate Leaf | F2-ID-139 | Lanceolate Leaf |
| F2-ID-92 | Obcordate Leaf | F2-ID-154 | Lanceolate Leaf |
| F2-ID-114 | Obcordate Leaf | F2-ID-13 | Lanceolate Leaf |
| F2-ID-107 | Obcordate Leaf | F2-ID-14 | Lanceolate Leaf |

^ǂ^Parents of F_2_ mapping population

**Table S6.** Chromosome wise SNPs distribution between early flowering (EF) and late flowering (EF) pools

| Pseudomolecule | LF  SNPs count | LF  homozygous SNPs count | EF  SNPs count | EF  homozygous SNPs count | LF  lower depth | LF  maximum depth | EF  lower depth | EF  maximum depth | SNP start position  (bp) | SNP end position  (bp) |
| --- | --- | --- | --- | --- | --- | --- | --- | --- | --- | --- |
| CcLG01 | 3795 | 654 | 3574 | 469 | 5 | 70 | 5 | 68 | 4550 | 17673307 |
| CcLG02 | 6902 | 1018 | 6895 | 1037 | 5 | 74 | 5 | 67 | 310 | 36917743 |
| CcLG03 | 5545 | 2464 | 4868 | 756 | 5 | 68 | 5 | 57 | 5602 | 29460718 |
| CcLG04 | 2926 | 430 | 2983 | 603 | 5 | 70 | 5 | 87 | 846 | 12497655 |
| CcLG05 | 866 | 122 | 846 | 106 | 5 | 32 | 5 | 37 | 7549 | 5132925 |
| CcLG06 | 4002 | 605 | 4137 | 662 | 5 | 64 | 5 | 68 | 42877 | 23783515 |
| CcLG07 | 3799 | 644 | 3856 | 681 | 5 | 73 | 5 | 73 | 5795 | 19558610 |
| CcLG08 | 4103 | 623 | 3931 | 550 | 5 | 53 | 5 | 50 | 1400 | 20381616 |
| CcLG09 | 2056 | 289 | 2032 | 276 | 5 | 77 | 5 | 61 | 2319 | 10417312 |
| CcLG10 | 4278 | 734 | 4242 | 723 | 5 | 66 | 5 | 58 | 1439 | 22568409 |
| CcLG11 | 9157 | 1655 | 9146 | 1564 | 5 | 82 | 5 | 73 | 438 | 48935041 |
| Total | 47429 | 9238 | 46510 | 7427 |  |  |  |  |  |  |

EF: Early flowering

LF: Late lowering

**Table S7.** Chromosome wise SNPs distribution between lanceolate leaf shape (LLS) and obcordate leaf shape (OLS) pools

| Pseudomolecule | OLS  SNPs count | OLS  homozygous SNPs count | LLS  SNPs count | LLS  homozygous SNPs count | OLS  lower depth | OLS  maximum depth | LLS  lower depth | LLS  maximum depth | SNP start position  (bp) | SNP end position  (bp) |
| --- | --- | --- | --- | --- | --- | --- | --- | --- | --- | --- |
| **CcLG01** | 4504 | 781 | 4405 | 588 | 5 | 73 | 5 | 65 | 22921 | 17673187 |
| **CcLG02** | 8297 | 1336 | 8093 | 1312 | 5 | 67 | 5 | 74 | 3437 | 36917743 |
| **CcLG03** | 6194 | 1240 | 5926 | 1024 | 5 | 64 | 5 | 70 | 3820 | 29458342 |
| **CcLG04** | 2977 | 487 | 2985 | 497 | 5 | 59 | 5 | 61 | 6617 | 12497655 |
| **CcLG05** | 960 | 147 | 981 | 149 | 5 | 68 | 5 | 56 | 7487 | 5130602 |
| **CcLG06** | 5079 | 1025 | 4620 | 727 | 5 | 74 | 5 | 84 | 816 | 23783515 |
| **CcLG07** | 4324 | 697 | 4319 | 665 | 5 | 83 | 5 | 73 | 5669 | 19571839 |
| **CcLG08** | 4716 | 1504 | 3376 | 720 | 5 | 128 | 5 | 70 | 1581 | 20378814 |
| **CcLG09** | 2209 | 335 | 2264 | 329 | 5 | 67 | 5 | 52 | 5744 | 10427618 |
| **CcLG10** | 4888 | 960 | 4663 | 924 | 5 | 65 | 5 | 67 | 1369 | 22570046 |
| **CcLG11** | 10408 | 2009 | 10026 | 1835 | 5 | 85 | 5 | 79 | 438 | 48935275 |
| **Total** | 54556 | 10521 | 51658 | 8770 |  |  |  |  |  |  |

LLS: Lanceolate leaf shape

OLS: Obcordate leaf shape

**Table S8.** Identification of SNPs in the identified genomic regions for days to flowering (attached as dataset in excel format)

Table S9. Identification of SNPs in the identified genomic regions for leaf shape (attached as dataset in excel format)

Table S10. GBS data (Gb) generated for F_2_ mapping population (ICP 5529 × ICP 11605) and features of the genetic map

| **Features**^†^ | **ICP 5529 × ICP 11605** |
| --- | --- |
| Total GBS data (Gb) | 30.15 |
| P_1_ | 0.543 |
| P_2_ | 0.163 |
| F_2_ - range | 0.041-0.531 |
| F_2_ - average | 0.168 |
| **Genetic map** |  |
| No. of total SNPs | 12662 |
| No. of SNPs showing severe segregation distortion (P<1.0x10^-9^) | 9727 |
| No. of markers segregating at 1:2:1 at P ≥ 1.0 x 10^-9^ | 2935 |
| No. of markers in anchor maps | 140 |
| Length of anchor maps | 584.2 |
| No. of total mapped loci | 787 |
| - Mapped non-distorted loci | 262 |
| - Mapped distorted loci | 525 |
| Total map length (cM) | 1454.0 |
| Average marker spacing (cM) | 1.8 |
| Number of gaps >10.0 cM | 21 |
| Largest gap (cM) | 25.4 |

^†^Information obtained from Saxena et al.^12^.

**Table S11.** Summary of main effect QTLs detected by composite interval mapping (CIM)

| QTL | Linkage group | Position | Left Marker | Left Position | Right Marker | Right Position | QTL-interval | LOD | Additive | Dominance | PVE (%) | Genes† |
| --- | --- | --- | --- | --- | --- | --- | --- | --- | --- | --- | --- | --- |
| *Days to flowering* | | | | | | | | | | | |  |
| ***qDF3.1*** | 3 | 24.71 | S3_22234078 | 23.7 | S3_16681929 | 29.4 | 5.7 | 7.81 | -6.00 | 4.75 | 39.58 | 588 |
| *qDF3.2* | 3 | 34.31 | s3_20698771 | 34.3 | S3_18430894 | 36.6 | 2.3 | 7.83 | 5.25 | 3.96 | 4.60 | 256 |
| ***qDF3.3*** | 3 | 41.61 | S3_18430894 | 36.6 | S3_18154848 | 43.6 | 7.0 | 5.03 | -5.57 | 5.47 | 47.58 | 29 |
| ***qDF3.4*** | 3 | 46.61 | S3_18154848 | 43.6 | S3_17193829 | 49.6 | 6.0 | 3.98 | -4.04 | 2.66 | 16.18 | 90 |

QTLs in bold face are major (Phenotypic variance explained; PVE ≥ 10 %)

^†^Total number of genes present in the QTL regions

**Table S12.** Comparison of the identified QTLs from QTL-seq with conventional QTL mapping

| QTLs | Trait | GBS | | QTL-seq |
| --- | --- | --- | --- | --- |
|  |  | Physical position | PVE (%) | Physical position |
| *qDF3.1* | DF | 16681929-22234078 (5.55 Mb) | 39.58 | 19222701-20806617 (1.58 Mb) |
| *qDF3.2* | DF | 18430894-20698771 (2.26 Mb) | 4.60 | 19222701-20806617 (1.58 Mb) |
| *qDF3.3* | DF | 18154848-18430894 (0.27 Mb) | 47.58 | - |
| *qDF3.4* | DF | 17193829-18154848 (0.96 Mb) | 16.18 | - |

DF; days to flowering; values in parenthesis indicate the length of the genomic regions mapped during the study

**Table S13.** Protein domain prediction and functional GO class identification through Interpro (attached as dataset in excel format).

**Table S14.** Sequence similarity match using DELTA-BLAST (attached as dataset in excel format)

**Table S15**. List of genes identified to have high collinear synteny along other *Fabaceae* genomes corresponding to the candidate genes (attached as dataset in excel format).
